# More than Meets the Eye: Functionally Salient Changes in Internal Bone Architecture Accompany Divergence in Cichlid Feeding Mode

**DOI:** 10.1155/2012/538146

**Published:** 2012-05-15

**Authors:** R. Craig Albertson, W. James Cooper, Kenneth A. Mann

**Affiliations:** ^1^Department of Biology, University of Massachusetts Amherst, 611 North Pleasant Street, Amherst, MA 01003, USA; ^2^School of Biological Sciences, Washington State University Tri-Cities, 2710 Crimson Way, Richland, WA 99354, USA; ^3^Institute for Human Performance, Department of Orthopedic Surgery, SUNY Upstate Medical University, 750 East Adams Street, Syracuse, NY 13210, USA

## Abstract

African cichlids have undergone extensive and repeated adaptive radiations in foraging habitat. While the *external* morphology of the cichlid craniofacial skeleton has been studied extensively, biomechanically relevant changes to *internal* bone architecture have been largely overlooked. Here we explore two fundamental questions: (1) Do changes in the internal architecture of bone accompany shifts in foraging mode? (2) What is the genetic basis for this trait? We focus on the maxilla, which is an integral part of the feeding apparatus and an element that should be subjected to significant bending forces during biting. Analyses of *μ*CT scans revealed clear differences between the maxilla of two species that employ alternative foraging strategies (i.e., biting versus suction feeding). Hybrids between the two species exhibit maxillary geometries that closely resemble those of the suction feeding species, consistent with a dominant mode of inheritance. This was supported by the results of a genetic mapping experiment, where suction feeding alleles were dominant to biting alleles at two loci that affect bone architecture. Overall, these data suggest that the internal structure of the cichlid maxilla has a tractable genetic basis and that discrete shifts in this trait have accompanied the evolution of alternate feeding modes.

## 1. Introduction

Adaptive radiations involve the concomitant evolution of ecological and phenotypic diversity within a rapidly multiplying lineage [1], and many of the most notable adaptive radiations are characterized by divergence in functional morphology. Hawaiian silverswords, for example, have evolved a suite of morphological traits associated with adaptations to an extreme range of environmental moisture (mesic to xeric) [2, 3]; *Anolis* lizards have diversified in regard to traits involved in clinging and climbing abilities [4–8]; both Galápagos finches and African cichlids are renowned for their extensive (and in the case of cichlids, repeated) adaptive radiations in trophic morphology that parallel, and presumably contribute to, microhabitat divergence in foraging niches [9–16]. Not surprisingly, considerable attention has been given to characterizing the phenotypic diversity associated with these extraordinary radiations [3, 4, 9, 14, 17–19].

 In the case of the multiple adaptive radiations of East-African cichlids, extensive analyses of their anatomical diversity have only recently been undertaken [14, 20, 21]. Among the notable findings from this body of work is that patterns of diversification within each of the three large lakes in the region (Victoria, Tanganyika, and Malawi) are statistically similar to one another [14, 21]. In particular, previous work from our group has found that trophic variation among cichlid radiations is characterized by divergence along a conserved ecomorphological axis [14]. One end of this axis is defined by species that forage in the water column and possess elongated jaws, while the opposite end is characterized by species that feed on benthic prey items using significantly shorter jaws. Thus, the primary axis of craniofacial variation defined by East-African rift-lake cichlids distinguishes benthic from pelagic ecotypes. The concordance between morphology and foraging mode observed in this study makes sense within the more general context of teleost functional morphology. Fish with short jaws have the potential, all other factors being equal, to produce bites that are proportionally more powerful due to an increased mechanical advantage employed by the jaw adductor muscles during biting, which is advantageous for herbivores that scrape tough, filamentous plant material from the substrate and for benthic predators that generate larger bite forces in order to crush, detach, or uncover their prey [22–25]. Longer jaws, on the other hand, facilitate the capture of more elusive prey by increasing bite speed and promoting greater jaw protrusion [23, 26–31].

While the functional implications of variation in external craniofacial geometry have been extensively studied in fishes [24, 28–30, 32–34] and many other vertebrates [18, 35–39], the examination of internal bone architecture has been less prominent with respect to adaptive radiations in fishes (but see [40, 41]). This paucity of data likely reflects the effort and expense associated with obtaining descriptions of these phenotypes. Specifically, while *μ*CT scanning is becoming increasingly accessible to more research labs and is therefore being applied to the study of a steadily increasing number of taxa, the collection and processing of this type of data remains time-consuming, computationally intensive, and expensive in comparison to imaging external bone shape (which may only require light photography). Since adaptive radiations, by definition, result in species-rich and/or highly diverse lineages, the scanning of large numbers of skeletons is simply not feasible for most labs. Here we mitigate these limitations by focusing on one of the more stalwart, and functionally relevant, bones in the face (the maxilla) and by taking advantage of our current knowledge of cichlid adaptive radiations. In particular, we focus our analyses on species that define opposite ends of the functional continuum that characterizes the primary axis of craniofacial variation among Lake Malawi cichlid species [14]. In this way we can identify and describe trends that are associated with the primary axis of diversification of this lineage as well as generate a predictive framework for more global patterns of functional divergence among cichlids and other fish species. We find that discrete changes in the internal architecture of the maxilla have accompanied shifts in foraging mode within this group. These anatomical changes are biomechanically relevant and predict that biting species possess bone that is more resistant to force transmission compared to pelagic suction feeders. Finally, we show that variation in this trait has a relatively simple genetic basis, which suggests that it can respond quickly to natural selection. We submit that a more comprehensive understanding of the genetic architecture and phenotypic variation of this functionally important trait should be a priority of future research in this and other adaptive radiations defined by divergence in feeding morphology.

## 2. Methods

### 2.1. Focal Species

 Two closely related Lake Malawi cichlid species that employ alternate modes of feeding (biting versus suction) were analyzed for this study. *Labeotropheus fuelleborni* (LF) is a member of the rock-dwelling clade of Malawi cichlids that is specialized to scrape tough, filamentous algae from the substrate [42, 43], and it has one of the most extreme craniofacial architectures of any lake-dwelling cichlid species within this region [14]. It possesses a short, stout head, steeply rounded craniofacial profile and wide jaws that are configured to employ high mechanical advantage during biting. *Maylandia zebra* (MZ; the genus name *Metriaclima*, which the authors have used elsewhere, is a junior synonym of *Maylandia*; [44]) is a closely related, but more generalized rock-dwelling species that collects plankton from the water column and brushes loose algae and detritus from rocky substrates. To accommodate this alternate mode of feeding, MZ has a relatively long head, shallow skull profile, and elongated jaws that are configured to produce faster but weaker bites (i.e., lower MAs) relative to LF [42, 43].

**Figure 1:**
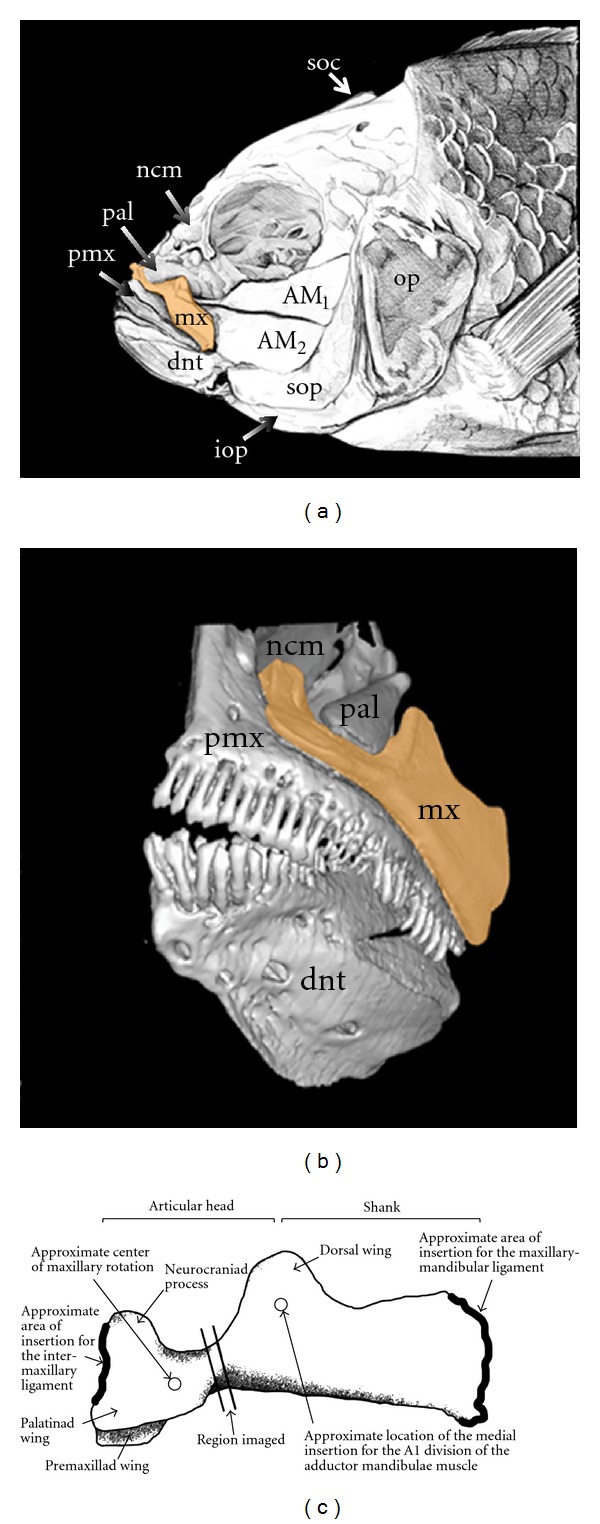
(a) Illustration of cichlid craniofacial anatomy in the lateral view. (b) Micro-CT scan of the oral jaws and associated elements. (c) Anatomy of a cichlid maxilla (left side, lateral view) showing the region imaged using *μ*CT scanning. In panels (a) and (b) the maxilla (mx) is highlighted orange. Drawing by Kristen Ann Tietjen. AM_1_: first division of the *adductor mandibulae* muscle; AM_2_: second division of the *adductor mandibulae*; dnt: dentary; iop: interopercle; ncm: neurocranium; op: opercle; pal: pterygoid process of the palatine; pmx: premaxilla; soc: supraoccipital crest of the neurocranium; sop: subopercle.

**Figure 2:**
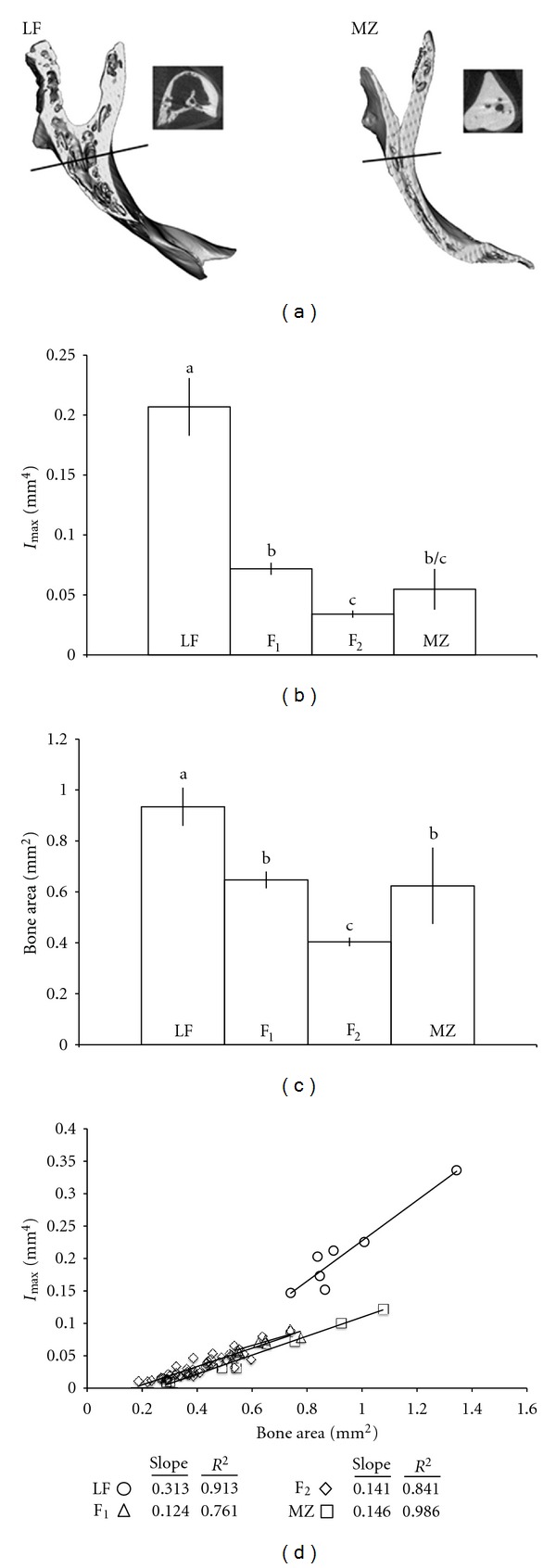
(a) LF and MZ exhibit clear differences in internal bone architecture. Both frontal and transverse sections are shown. Frontal sections were taken approximately halfway through the bone. Lines through the elements show the level at which transverse sections were taken. Differences in bone architecture were quantified as bone bending stiffness (i.e., *I*_max⁡_ mm^4^, (b)) and bone cross-sectional area (mm^2^, (c)). For both measures, the F_1_ and F_2_ hybrid generations are intermediate, with a statistical bias toward MZ values. For both (b) and (c), the “a, b, and c” indicate statistical groupings according to a two-tail *t*-test, and bars indicate standard errors. (d) Linear regression of bone area on bending stiffness. The relationship between bone bending stiffness and area is approximately the same for MZ and both hybrid generations, but different for LF, which are more efficient in terms of generating greater bending stiffness via the distribution of bone.

We have shown previously that the forces generated during biting will be transmitted from the lower jaw, through the maxillae, and to the anterior portions of the neurocranium and palatine ([34], Figure 1). Bending force load should also be high in the maxilla, since it acts as a lever that pivots around the pterygoid process of the palatine (Figure 1), and which is moved by the A1 division of the *adductor mandibulae* muscle during biting and by its connection to the lower jaw during mouth opening [32]. The shape of the maxillae is conspicuously different in LF and MZ (Figures 1 and 2(a)), with LF possessing an element that is much wider and more conspicuously bent along the medial-lateral axis compared to MZ, where the maxilla is thin and straight. See Albertson and Kocher [42] for a more comprehensive discussion of the craniofacial anatomy of these two species and Otten [45] and Cooper et al. [34] for a description of the functional anatomy of the cichlid oral jaws.

### 2.2. Microcomputed Tomography and Quantification of Internal Bone Architecture

Maxillae were scanned at 12-micron resolution with a microcomputed tomography (*μ*CT) scanner (*μ*CT 40, SCANCO Medical, Wayne, PA). Maxillae were oriented for scanning such that cross-sectional images were perpendicular to the long axis of the articular head of the bone (Figure 1(c)). These cross-sections were taken through the thinnest portion of the caudal (i.e., “neck”) region of the articular head of the maxilla. This region is roughly semicircular in cross-section (Figure 2), and is caudal to the maxilla's premaxillary and palatinad wings and rostral to its dorsal wing (anatomy after [46]). It lies between the articulation of the palatinad wing of the maxilla with the maxillad process of the palatine (the fulcrum for maxillary rotation) and the two regions where closing and opening forces are applied to the maxilla: the insertion of the A1 division of the *adductor mandibulae* muscle on the medial surface of the dorsal wing (closing) and the ligamentous attachments between the shank of the maxilla and the lower jaw (opening). The maxillary shank is connected to the lower jaw by connective tissue that attaches to both the primordial process of the articular and the coronoid process of the dentary in the fishes we examined (the maxillary connection to the dentary is sometimes less extensive in other fish species). The cross-sectional areas imaged lie almost immediately between the maxillary fulcrum and the point where biting (i.e., closing) forces are directly applied to this bone (Figure 1(c)), and an ability to resist bending should therefore be a particularly important aspect of the functional morphology of this region. Image sets were exported to ImageJ (http://rsbweb.nih.gov/), and a lower threshold was defined as 400 mg/cc hydroxyapatite equivalent to isolate bone. The BoneJ plug-in to ImageJ was used to quantify bone cross-sectional area (CSA, mm^2^) and principal area moment of inertia (*I*_max⁡_, mm^4^) of a single 2D slice within the articular neck of the maxilla (bracketed region, Figure 1(c)). CSA is a measure of the quantity of bone while *I*_max⁡_ is a measure of the ability of bone to resist bending loads. In all, 7 LF, 7 MZ, 7 F_1_, and 49 F_2_ were scanned and analyzed in this study. 

### 2.3. Pedigree and Linkage Analysis

 Details concerning the mapping population, construction of the linkage map, and quantitative trait locus (QTL) analysis have been described elsewhere [13, 34, 47, 48]. In brief, we used a pedigree derived from crossing a single LF male to a single MZ female to generate an F_2_ mapping population (*n* = 173) and a linkage map that assigned 165 markers (both microsatellites and SNPs) to 25 linkage groups using JoinMap 3.0 [49]. A linkage analysis was performed using MapQTL 4.0 [50] with *I*_max⁡_ as the mapping variable. Because of the time and expense required to *μ*CT scan cichlid maxillae, we chose 49 F_2_ animals with a wide range of external maxillary thicknesses for our QTL analysis.

It is important to note that, while our experimental design (i.e., bulk segregants) captured much of the variance in maxillary width among our F_2_, the relatively small number of F_2_ used in this experiment makes the results susceptible to the Beavis effect, in which the number of QTL tends to be underestimated and QTL effects tend to be overestimated [51]. These specific variables should therefore be interpreted with caution as they likely represent a simplified view of the genetic architecture of these traits. However, both modeling and empirical data indicate that the accuracy of QTL localization is less affected by small sample sizes [52, 53]. Nevertheless, we consider this analysis to be largely a proof of concept and the results to be preliminary. 

## 3. Results

### 3.1. Distinct Internal Bone Architectures Are Associated with Divergent Feeding Modes

Micro-CT scanning revealed clear qualitative differences in internal bone morphology between LF and MZ (Figure 2(a)). We found that the maxilla in LF is hollow, with an internal bone architecture that closely resembles that of trabecular bone in mammals. The maxilla of MZ, on the other hand, is comparatively thin and solid. We reported previously that skull bone hydroxyapatite (HA) densities are roughly similar between these two species (LF: 708 ± 100 mg/cc HA; MZ: 757 ± 130 mg/cc HA; [34]), which suggests that any difference in biomechanical performance should be due to geometry rather than substance. To this end, we quantified differences in bone area moment of inertia (i.e., estimated bending stiffness, *I*_max⁡_ mm^4^, Figure 2(b)) and bone cross-sectional area (CSA, mm^2^, Figure 2(c)). We found that the maxilla in LF contains significantly more bone than MZ and is also significantly more resistant to bending forces. Moreover, the relationship between bending stiffness and CSA suggests that the internal architecture of the maxilla in LF is more structurally stalwart per unit of bone compared to MZ. This assertion is supported by a steeper slope describing the relationship between bone stiffness and area in LF compared to that in MZ (Figure 2(d)). For both measures, the F_1_ and F_2_ hybrid generations were statistically biased toward MZ, suggesting a role for dominance in the inheritance of these traits. Moreover, the relationship between bending stiffness and CSA was approximately the same for the MZ, F_1_ and F_2_ populations.

### 3.2. Genetic Architecture of a Biomechanical Trait

Two significant QTL were detected for bone bending stiffness (Table 1). The first (*I*_max⁡_ 1) localized to a narrow region on linkage group 7. The second QTL (*I*_max⁡_ 2) localized to the distal end of linkage group 11. Both loci showed evidence for dominance of the MZ allele, which is consistent with the mean values for each population reported in Figure 2. The LF/LF genotype increased mean bending stiffness at both loci, although the mean phenotypic values of all genotypic classes were lower than what would be expected based on parental averages. This is likely due, at least in part, to our low F_2_ sample size, which has also likely acted to inflate the percent variance explained (PVE) by each QTL. We cannot, however, rule out the possibly that other factors are leading to a downward bias in our F_2_ values of stiffness, including environmental effects, or allometry. While we made every attempt to maintain constant rearing conditions across populations in terms of tank densities, substrate type, and diet, the F_2_ were raised a couple of years after the parental and F_1_ populations making it possible that there were unaccounted for differences in environment. Allometry could also be biasing our F_2_ values. The average size of our F_2_ population was smaller than that for either parental species or the F_1_ (average standard length of 8.0 cm (F_2_) versus 9.2 cm (LF), 9.3 cm (MZ), and 8.8 cm (F_1_)). However, when using residuals from a regression of stiffness on size, the QTL results did not change. Clearly, this observation warrants further investigation.

We chose F_2_ individuals for this analysis that exhibited a wide range of maxillary widths, with the intention of maximizing variance and thus the power to detect QTL. However, once these elements were scanned and stiffness was estimated, we found that width was only a weak predictor of bone stiffness (*R* ^2^ = 0.087, *P* = 0.121). In other words, these traits are segregating largely independent of one another, which suggests that they are under separate genetic control and that external skeletal anatomy cannot predict internal bone architecture. This assertion is supported by the observation that neither of the bending stiffness QTL fell within intervals that were previously implicated in maxillary shape [13]. In fact, QTL *I*_max⁡_ 2 localized to a region that is distinct from all other cichlid craniofacial QTL identified to date [13, 34, 48, 54, 55]. QTL *I*_max⁡_ 1, on the other hand, did localize to a region that overlaps with a QTL for jaw width [13] and exhibits a nearly identical LOD distribution with a QTL for the length of the retroarticular (RA) process of the lower jaw [48]. Similar to QTL *I*_max⁡_ 1, LF alleles at this locus act to increase the trait value for RA length and MZ alleles appear to be dominant.

## 4. Discussion

### 4.1. Divergence in Bone Strength and Weight among Vertebrates

Bone strength and stiffness are critical for optimizing the function of skeletal elements associated with feeding and locomotion, and natural selection will favor animals that perform these functions with greater efficiency [56–58]. While both bone density and shape contribute to stiffness and strength, dense bone is heavier than less dense bone. Vertebrate bone therefore tends to be designed such that strength and stiffness are maximized and weight is minimized [58, 59]. This trade-off is especially important in flighted vertebrates, where skeletons must be lightweight to minimize the metabolic cost of flight but strong enough to withstand the torsion and shearing forces associated with powered flight. As a result, birds and bats have evolved bones that are hollow but more dense compared to those of terrestrial vertebrates [60–62].

 There is also a dynamic relationship between bone strength and weight among aquatic vertebrates. Specifically, across a spectrum of vertebrate classes the modulation of bone density appears to be a mechanism for buoyancy control [63–65]. This trend is beautifully illustrated by the evolutionary history of whales, which is marked by discrete shifts in habitat from terrestrial, to semiaquatic, and finally to fully aquatic life histories. The mechanical constraints associated with locomotion in each of these habitats are very different, and as a result these evolutionary transitions were accompanied by dramatic changes in bone architecture. For example, the shift from terrestrial to semiaquatic habitats in ancient whales (i.e., archaeocetes) was accompanied by a dramatic increase in bone density. Like other large semiaquatic mammals, this adaptation was for increased mass, which is associated with benthic foraging [63]. Modern whales, on the other hand, are fully aquatic and possess a number of adaptations for life in the open water, including a largely osteoporotic skeleton [63]. While functional parameters including bone stiffness have not been examined in modern cetaceans, it is notable that osteoporotic bone in cetaceans is not observed in elements associated with feeding or locomotion (i.e., skull and vertebrae), where functional demands remain high [64]. Thus, a balance has been struck between increased buoyancy and efficient foraging and locomotion in the skeletons of modern whales.

The evolutionary history of Antarctic notothenioid fishes represents another striking example of how bone development has been modified to affect buoyancy. Antarctic notothenioids represent one of the best described adaptive radiations among marine fishes [66], and the hallmark of their evolution is the development of secondary pelagicism via alteration of buoyancy [67]. This lineage is thought to have evolved from a robustly mineralized bottom-dwelling perciform species beginning 40–60 mya when the waters of the Antarctic continental shelf were still temperate [67]. The grounding of the ice sheet on the continental shelf and changing trophic conditions led to the local extinction of the diverse late Eocene fish fauna, thus freeing pelagic niches into which the notothenioids radiated [68]. About 50% of notothenioid species now either live or forage in the pelagic habitat [69]. In many instances, secondary pelagicism has been achieved through pedomorphism, including the complete or partial retention of the notochord, delayed ossification of the skeleton, and replacement of bone by connective tissue [65, 70–72]. Similar to cetaceans, osteoporotic bone in pelagic notothenioids is most pronounced in areas of the skeleton that are not intimately associated with foraging (e.g., oral jaws) or locomotion (e.g., pectoral fins) [70].

While the examples above represent changes in bone structure at the macroevolutionary level, it is reasonable to assume that similar trends underlie microevolutionary divergence. As mentioned above, cichlids have diverged along a benthic-pelagic ecomorphological axis, and extensive modifications to the skeletal system have accompanied this divergence [14]. LF and MZ are closely related species that lie on opposing ends of this continuum, and while bone density does not appear to be different between these two species [34], LF has a more extensively mineralized skeleton (i.e., more bone in more places) [42], which is commensurate with other adaptations for a benthic mode of feeding. These findings suggest that levels and patterns of bone deposition are more evolvable in this group than are the material properties of bone (although a more rigorous survey of HA density in a greater number of elements and across more taxa is needed). We also show here that internal bone architecture appears to be surprisingly malleable among cichlids, as strikingly different cross-sectional bone shapes exist between species that employ alternate modes of feeding. This sets up clear predictions that can be tested in a larger number of species. For example, if species were arrayed along a benthic-pelagic ecomorphological axis, one might expect that this would establish a continuum of internal bone architectures. Alternatively, since LF represents a highly derived species, it is also possible that the internal bone architecture described here (i.e., high stiffness) is unique to this species. Clearly, this would be a fruitful area of future research.

### 4.2. Roles for the Environment versus Genetics in Determining Internal Bone Architecture

Bone geometry influences stiffness such that bone with a solid cross-section is less rigid whereas hollow bone with the same cross-sectional area is more rigid. Natural selection should therefore favor one configuration over the other depending on the task to be performed (e.g., biting versus sucking). Alternatively, given the varying functional demands imposed on the vertebrate skeletal system over ontogeny, or from season to season, selection might favor a plastic skeletal system that can respond to different mechanical stimuli. Distinguishing between these alternatives represents an important, but muddled area of research. In other words, the degree to which internal bone architecture is genetically preprogrammed or mechanically regulated remains unclear.

 On one hand, both computational modeling and empirical studies offer strong support for the assertion that internal bone architecture responds to mechanical stimuli [59, 73, 74]. Alternatively, disparate vertebrate taxa have modified internal bone geometry due to novel functional demands (e.g., powered flight in birds and bats) [35, 61, 62], which suggests a genetic component for this trait. Unfortunately, compared to the relatively large body of literature dedicated to the study of the genetics of bone material properties (focused mainly on mouse mutants, reviewed by [75]), less is known about the genetic basis of bone geometry. Moreover, mutations that lead to aberrant bone architectures usually also affect material properties. “Wolff's Law” suggests that bone adapts to mechanical stimuli to maintain a narrow range of strain (reviewed by [76]). It is therefore thought that for many/most mouse mutants where both bone material and geometry are affected, deficient material properties are the primary defect and altered geometry represents a secondary response to compensate for abnormal bone strains [76].

Cichlids offer a genetic system where internal bone architecture varies independently from material properties, thus mitigating the confounding issues associated with Wolff's Law. Whereas HA density appears relatively conserved between the species examined here, internal geometry differs dramatically. This could be due to a fundamental constraint associated with changing material properties in fishes (e.g., higher-density bone is more brittle) or because altering bone architecture is a more efficient way to affect stiffness. For example, bending stiffness of a round bone is equal to *EI*, where the elastic modulus (*E*) is proportional to HA density of bone and area moment of inertia (*I*) is proportional to radius^4^. Doubling HA would lead to a doubling of stiffness, whereas doubling the radius would lead to a 16-fold increase in stiffness. Changing bone architecture is therefore a more efficient way to change bone function due to increased demands on bending load. Either way, the decoupling of these two properties of bone, as well as the ability to perform genetic mapping studies, offers an excellent opportunity to examine the genetic basis of internal bone geometry. Moreover, the ability to rear cichlids on a range of diets (e.g., hard versus soft), thereby altering the mechanical environment in which the jaws develop, would enable an assessment of the degree to which this trait responds to the environment. Thus, cichlids represent an ideal system in which to characterize the genetic and environmental factors that influence this functionally salient trait.

### 4.3. Conclusions

We demonstrate that cichlids with divergent feeding morphologies and behaviors exhibit different internal bone architectures that translate to different estimates of load-bearing function. We show further that this functional trait has a tractable genetic basis. Since bone geometry has a profound effect on skeletal performance and since performance determines resource use in nature, examining the genetic basis for this trait has the potential to yield important new insights into the mechanisms that have contributed to several notable adaptive radiations (e.g., those that involve divergence in feeding systems or locomotion). Moreover, continued work in the cichlid system may also contribute to an understanding of many unresolved issues in the biomedical literature, especially those focused on decoupling the genetic from epigenetic influences on internal bone geometry. In conclusion, external bone shape and size, while important in determining skeletal function, do not tell the entire story [76]. Future research should therefore be aimed at elucidating a better understanding of (1) the material properties and (2) internal geometry of skeletal elements associated with feeding and locomotion in this and other adaptive radiations.

## Figures and Tables

**Table 1:** Two distinct QTL on two linkage groups (LGs) were detected for bone bending stiffness. Both loci show evidence for dominance of the MZ allele, which is consistent with the mean values for each population reported in Figure 2. The LF/LF genotype increases mean stiffness at both loci, although the mean phenotypic values of each genotypic class were lower than what would be expected based on parental averages. This is likely due to our low F_2_ sample size, which has also likely inflated the percent variance explained (PVE) by each QTL.

Trait	Parental means (SE)		95% range* peak				Mean phenotype/F_2_ genotype				
	MZ	LF	QTL	LG	cM	cM	LOD	MZ/MZ	MZ/LF	LF/LF	PVE
Stiffness [*I*_max⁡_ (mm^4^)]	0.055 (0.017)	0.207 (0.024)	*I*_max⁡_ 1	7	51–57	54	3.80	0.0326	0.0361	0.0606	23.4
			*I*_max⁡_ 2	11	49–50	50	3.10	0.0298	0.0378	0.0630	38.5

*Significance (*α* = 0.05) at the genomewide level.
